# Systematic Review and Meta‐Analysis of Mismatch Negativity in Autism: Insights Into Predictive Mechanisms

**DOI:** 10.1002/aur.70131

**Published:** 2025-10-30

**Authors:** Laurie‐Anne Sapey‐Triomphe, Romain Bouet, Jérémie Mattout, Sandrine Sonié, Christina Schmitz, Françoise Lecaignard

**Affiliations:** ^1^ Université Claude Bernard Lyon 1, CNRS, INSERM Centre de Recherche en Neurosciences de Lyon CRNL U1028 UMR5292, CAP ‐ EDUWELL ‐ COPHY (INRIA Lyon) Teams Bron France; ^2^ Centre de Ressource Autisme Rhône‐Alpes Centre Hospitalier Le Vinatier Bron France

**Keywords:** adaptation, auditory, Autism Spectrum Disorders, EEG, mismatch negativity (MMN), perceptual learning, predictive coding

## Abstract

Mismatch negativity (MMN) has been frequently used to assess auditory processing and change detection in autism spectrum disorder (ASD), but findings have been fairly inconsistent. To address this issue, we conducted a systematic review and meta‐analysis of MMN amplitude (76 effect sizes) and latency (62 effect sizes) in ASD to identify factors contributing to this heterogeneity and to interpret findings within the predictive coding framework. While residual heterogeneity remained, significant effects of the interaction between age group and design type (unifeature vs. multifeature, i.e., one or several types of deviants) and deviant type were found for MMN amplitude. In multifeature designs, autistic children and adolescents exhibited reduced MMN amplitudes compared to neurotypical peers (*g* = 0.25, *p* = 0.01), whereas autistic adults showed increased MMN amplitudes (*g* = −0.26, *p* = 0.02). In addition, autistic individuals had significantly smaller MMN amplitudes than neurotypical individuals in paradigms using phoneme deviants (*g* = 0.41, *p* < 0.001). Across designs, no significant MMN latency differences were observed between neurotypical and autistic individuals. These results are discussed within the predictive coding framework, as MMN responses are thought to reflect prediction errors, aligning with theories suggesting heightened prediction errors in autistic adults. Future studies with larger samples and improved data reporting are needed to further clarify the developmental trajectory and variability of MMN responses in ASD. Additionally, computational modeling approaches can help characterize learning dynamics and disentangle predictive coding accounts among autistic individuals.

**Summary**

- The mismatch negativity (MMN) is a brain response triggered by a change or irregularity in a sequence of sensory stimuli.
- As such, it is expected to shed light on how autistic individuals process and adapt to unexpected auditory changes.
- This meta‐analysis, focusing on the auditory MMN in autism, reveals group differences in uncertain contexts, with different patterns for children and for adults.

**Abbreviations**
ASD: autism spectrum disorder
CI: confidence interval
HIPPEA: high and inflexible precision of prediction error in autism
MMN: mismatch negativity
NT: neurotypical

## 1 Introduction

Unexpected sounds trigger surprise, even in sleeping neonates (Sylvester et al. 2021), highlighting that the brain forms expectations and learns statistical regularities in its environment (Friston 2010; Rao and Ballard 1999). Predicting upcoming stimuli reduces uncertainty and facilitates optimal adaptation (Friston 2010). Within the predictive coding framework, perception amounts to learning statistical regularities through (Bayesian) inference. Perceptual learning and inference yield internal representations, also called predictions or “priors,” which are continuously confronted with the current state of the world. Mismatches between sensory inputs and priors generate prediction errors. The handling of these prediction errors is crucial: depending on their assigned weight, they can either update priors or be downweighted if they reflect irrelevant variability. Appropriately downweighting prediction errors is essential, as every experience is inherently different. In contrast, signaling every slight deviation between expectations and sensory inputs could result in constant surprise, overwhelming sensory processing, and ultimately increasing anxiety. Since perception is shaped by both predictions and prediction errors, this may lead to atypical perceptual experiences. This may be the case in autism, where atypical predictive mechanisms are hypothesized to underlie the symptoms (Pellicano and Burr 2012; Brock 2012; Sinha et al. 2014; Lawson et al. 2014; Palmer et al. 2017; Van de Cruys et al. 2014; Gomot and Wicker 2012).

Autism spectrum disorder (ASD) is a neurodevelopmental disorder characterized by deficits in social communication and interaction, restricted interests, repetitive behaviors, and atypical sensory responsivity (American Psychiatric Association 2013). Additionally, autistic individuals often exhibit heightened intolerance of uncertainty, a need for sameness, and a strong resistance to change (American Psychiatric Association 2013). According to predictive coding theories of ASD, autistic symptoms could be explained by atypical perceptual inference and/or learning (Haker et al. 2016). Two theories suggest that autistic individuals have a very “realistic” perception, either because they are barely influenced by prior knowledge (hypo‐prior theory (Pellicano and Burr 2012)) or because their sensory precision is overly high (high sensory precision theory (Brock 2012)). Another influential theory, the High and Inflexible Precision of Prediction Errors in Autism (HIPPEA) theory (Van de Cruys et al. 2014), suggests that autistic people are continuously overwhelmed by prediction errors. While the hypo‐prior theory implies difficulties in learning statistical regularities, HIPPEA posits that ASD reflects a stronger weight of prediction errors, which can either facilitate or hinder statistical learning, depending on the context.

Since the emergence of predictive coding theories of ASD, about a decade ago, empirical studies have tested their validity (for reviews, see (Cannon et al. 2021; Angeletos Chrysaitis and Seriès 2023)). Research has demonstrated that autistic individuals are capable of learning statistical regularities and are influenced by priors (Cannon et al. 2021; Angeletos Chrysaitis and Seriès 2023), challenging the simplistic hypo‐prior hypothesis (Pellicano and Burr 2012). However, findings remain mixed, with studies showing intact (Pell et al. 2016; Ego et al. 2016; Croydon et al. 2017; Utzerath et al. 2019), reduced (Sapey‐Triomphe, Weilnhammer, and Wagemans 2021; Karaminis et al. 2016; Król and Król 2019) or increased (Sapey‐Triomphe et al. 2018) prior influence in ASD depending on the context. More subtle differences include that autistic individuals show difficulties in flexibly updating priors (Sapey‐Triomphe, Weilnhammer, and Wagemans 2021; Sapey‐Triomphe, Timmermans, and Wagemans 2021; Lieder et al. 2019), estimating the volatility of the environment (Lawson et al. 2017), or adjusting the weight of prediction errors (Goris et al. 2018). Such group differences might especially emerge in uncertain or dynamically changing contexts.

Long before predictive coding theories of ASD emerged, research on ASD frequently measured mismatch negativity (MMN) responses to unexpected sounds to investigate auditory processing, attention, and memory (Schwartz et al. 2018; Chen et al. 2020). Interestingly, MMN is observed using oddball paradigms, which are ideally suited for testing predictive coding (Heilbron and Chait 2018; Fitzgerald and Todd 2020). Oddball sequences include repeated sounds (*standards*) that enable the learning of regularities, as well as unexpected or surprising sounds (*deviants*) that elicit prediction errors. MMN is now widely interpreted as reflecting such an error, that is, mismatches between top‐down predictions and bottom‐up sensory inputs (Friston 2005). Indeed, MMN responses arise whenever a comparison between the current stimulus and the memory trace of previous stimuli reveals a change in regularity (Giard et al. 1990; Dürschmid et al. 2016; Sedley et al. 2016). Interestingly, MMN is an automatic response that does not require any instructions but can be modulated by attention (Näätänen et al. 2001; Näätänen et al. 2012). It is therefore possible to measure MMN across a wide range of participants (Garrido et al. 2009; Morlet and Fischer 2014). Thus, MMN provides a unique window into prediction errors (Garrido et al. 2009), offering valuable insights into predictive coding mechanisms in ASD.

The MMN is an electrophysiological component computed as the difference in brain responses to standard (i.e., expected) and rare deviant (i.e., less expected) stimuli (Garrido et al. 2009; Näätänen et al. 1978; Näätänen et al. 2007). In typical oddball paradigms, deviance is obtained by changing the physical attributes of the standard sound (e.g., frequency or duration). Oddball paradigms are classified as unifeature or multifeature. Unifeature paradigms include one type of deviant (e.g., 90% standard, 10% deviant in duration), whereas multifeature paradigms involve multiple deviants (e.g., 70% standard, 10% deviant in duration, 10% deviant in frequency, 10% deviant in intensity). Multifeature designs introduce greater uncertainty due to variability across stimulus dimensions and a lower probability of the standard stimulus. Both deviance magnitude and occurrence modulate MMN amplitude, reflecting the magnitude of prediction error and the level of surprise, respectively. The auditory MMN manifests as a negative deflection that typically occurs in fronto‐central regions between 100 and 250 ms after stimulus onset (Garrido et al. 2009; Sams et al. 1985). It is detectable using electroencephalography (EEG) (e.g., Schwartz et al. 2018; Chen et al. 2020) or magnetoencephalography (MEG) (e.g., Lecaignard et al. 2021). MMN relies on a hierarchical organization of brain regions including the primary auditory cortices, superior temporal gyri, and inferior frontal gyri (Lecaignard et al. 2021; Garrido et al. 2007; Garrido et al. 2008). This cortical network involves both forward (bottom‐up) and backward (top‐down) connections, conveying prediction errors and predictions, respectively (Garrido et al. 2007; Garrido et al. 2008; Lecaignard et al. 2022). Network connectivity can be modulated by deviant probability, as predicted by the predictive coding framework (Lecaignard et al. 2022).

According to the predictive coding hypotheses (Pellicano and Burr 2012; Brock 2012; Van de Cruys et al. 2014), differences in MMN are expected in ASD. If MMN amplitude reflects the weight of prediction errors, the HIPPEA theory would predict larger MMN amplitudes in ASD, whereas the hypo‐prior theory would predict lower MMN amplitudes (i.e., as a low prior precision would lead to a decreased prediction error precision). MMN studies in ASD yielded mixed findings, with some studies indicating either reduced, typical, or increased MMN amplitude and latency in ASD (Schwartz et al. 2018; Chen et al. 2020). Two meta‐analyses were conducted to help draw conclusions from these heterogeneous results (Schwartz et al. 2018; Chen et al. 2020). The first meta‐analysis (Schwartz et al. 2018) mostly focused on comparisons between speech and non‐speech stimuli and found reduced MMN amplitude in young autistic children compared to neurotypical (NT) children in experiments that used nonspeech sounds. The second meta‐analysis (Chen et al. 2020) mostly assessed the influence of deviants in frequency, duration, and phoneme on MMN responses in ASD. They found that autistic individuals had reduced MMN amplitudes and prolonged latencies for deviants in phoneme, and that autistic children and adolescents had decreased MMN amplitudes for deviants in duration (Chen et al. 2020).

The present meta‐analysis aims to investigate MMN in ASD through the lens of predictive coding theories to shed light on perception in ASD. By adopting this framework, we can focus on paradigm features that are particularly relevant to predictive mechanisms, such as environmental uncertainty. In the predictive coding framework, uncertainty can take different forms, including expected uncertainty, which refers to the known variability within a stable context (Yu and Dayan 2005). In MMN paradigms, two factors reflect this form of uncertainty: the probability of deviant occurrence (i.e., how frequently a deviant stimulus appears) and the number of deviant types (e.g., deviants can differ in pitch, duration, intensity). Lower deviant probabilities allow for more precise predictions, and therefore, lower uncertainty. Paradigms with multiple deviant types increase the complexity of the environment and uncertainty. Although these two features are often interdependent, they can vary differently across studies. Investigating how these aspects of uncertainty modulate MMN responses is important, especially in light of theories suggesting different weights to prediction errors depending on contextual uncertainty in ASD and empirical data showing that uncertainty can either impair or preserve statistical learning in ASD (e.g., Sapey‐Triomphe, Weilnhammer, and Wagemans 2021; Sapey‐Triomphe, Pattyn, et al. 2023). These key experimental parameters may help explain some of the inconsistencies in MMN findings in ASD. In addition, consistent with the most recent meta‐analysis (Chen et al. 2020), the effect of deviant type on MMN was also investigated. Thus, this meta‐analysis seeks to clarify how specific paradigm features contribute to the heterogeneity observed in MMN studies of ASD. Moreover, this meta‐analysis expands upon previous meta‐analyses, as 14 new articles encompassing a total of 35 effect sizes were published since the last meta‐analysis. By integrating these new studies and analyzing MMN within a predictive coding framework, our findings will offer novel insights into prediction error weighting and statistical learning in ASD. Results are discussed within a predictive coding perspective.

## 2 Methods

### 2.1 Literature Search and Screening

**FIGURE 1:**
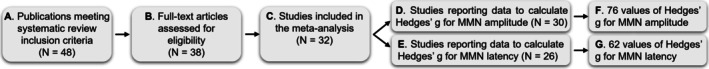
Flow chart of study selection for review and meta‐analysis on auditory MMN in ASD. (A) Published literature was searched in PubMed using the keywords [“mismatch negativity” OR “MMN”] AND [“autism” OR “ASD” OR “Asperger”] in the title and/or abstract. (B) Ten articles were excluded (see Section 2), and the remaining 38 articles are listed in Table 1 and discussed in the manuscript. (C) The final selection for the meta‐analysis included only EEG studies reporting MMN latency and/or amplitude. Four articles were excluded for not reporting means and standard deviations for both MMN amplitude and latency, and two were excluded for using MEG instead of EEG. (D, E) Studies were further selected if they reported both the mean (or difference in means) and the standard deviations of the MMN. (F, G) There are more Hedges' *g* values than articles, as many articles reported MMN responses under various experimental conditions. *N* indicates the number of articles included at each step.

The published literature was searched on PubMed using the keywords “mismatch negativity” or “MMN” or “MMF” and “autism” or “ASD” or “Asperger” in the title and/or abstract. Articles published up to July 2024 were included. Studies were considered eligible if they met the following inclusion criteria: (i) the publication reported an oddball EEG or MEG study involving autistic participants (i.e., with a clinical ASD diagnosis), (ii) the stimuli were presented in the auditory modality (modality largely prevalent in the MMN literature), (iii) the article was written in English. The following exclusion criteria were applied: (i) the study did not include neurotypical participants as a control group (*n* = 2), (ii) the article had received an expression of concern (*n* = 1), (iii) no significant MMN response was observed (*n* = 1), (iv) the study focused on brainstem measurements (*n* = 1), (v) the study employed a within‐group design only (*n* = 3). Table 1 summarizes the 38 articles that met these inclusion and exclusion criteria. The screening process is illustrated in Figure 1.

**TABLE 1:** Characteristics of the studies included in the review on auditory MMN in ASD.

Authors	Study name in meta‐analysis	*N*		Age (years)		EEG or MEG	MMN electrode	Experimental design						MMN in ASD	
		NT	ASD	NT	ASD			Design	Stimuli	P(std)	P(dvt)	Std/dvt	Condition	Latency	Amplitude
Gomot et al. (2002)	Gomot 2002	15	15	6.8	6.7	EEG	Fz	Uni.	Tone	85	15	1000/1100 Hz	Frequency	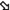	Ns
Ferri et al. (2003)	Ferri 2003	10	10	12.2	12.3	EEG	Fz	Uni.	Tone	80	10	1000/1300 Hz	Frequency	Ns	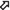
Jansson‐Verkasalo et al. (2003)	Jansson 2003a	11	10	9.6	9.1	EEG	F4, C4, P4, T4	Uni.	Tone	90	10	1000/1100 Hz	Frequency	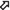	Ns
	Jansson 2003b							Uni.	Speech	80	20	≠ consonants	Phoneme	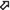	Ns
Jansson‐Verkasalo et al. (2005)	Jansson 2005	18	19	10.4	10.6	EEG	C3, C4, F4, P3, P4	Uni.	Tone	85	15	280/320 Hz	Frequency	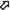	Ns
Kasai et al. (2005)	Not included	19	9	27.3	27.2	MEG	Mean over 28 to 43 channels	Uni.	Tone	90	10	100/50 ms	Duration	Ns	Ns
	Not included							Uni.	Speech	90	10	150/100 ms	Duration	Ns	Ns
	Not included							Uni.	Speech	90	10	≠ vowels	Phoneme	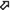	Ns
Lepistö et al. (2005)	Lepisto 2005a	15	15	9.4	9.4	EEG	Fz	Multi.	Speech	76	8	190/104 ms	Duration	Ns	Ns
	Lepisto 2005b										8	113/125 Hz	Frequency	Ns	Ns
	Lepisto 2005c										8	≠ vowels	Phoneme	Ns	Ns
	Lepisto 2005d	15	15	9.4	9.4	EEG	Fz	Multi.	Tone	76	8	190/104 ms	Duration	Ns	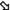
	Lepisto 2005e										8	113/125 Hz	Frequency	Ns	Ns
	Lepisto 2005f										8	Tone/Vowel	Phoneme	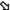	Ns
Lepistö et al. (2006)	Lepisto 2006a	10	10	8.1	8.1	EEG	Fz	Multi.	Speech	76	8	113/125 Hz	Frequency	Ns	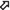
	Lepisto 2006b										8	190/104 ms	Duration	Ns	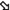
	Lepisto 2006c										8	≠ vowels	Phoneme	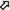	Ns
	Lepisto 2006d	10	10	8.1	8.1	EEG	Fz	Multi.	Tone	76	8	113/125 Hz	Frequency	Ns	Ns
	Lepisto 2006e										8	190/104 ms	Duration	Ns	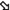
	Lepisto 2006f										8	Tone/Vowel	Phoneme	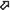	Ns
Korpilahti et al. (2007)	Korpilahti 2007	13	14	10.8	11.2	EEG	F3, F4	Uni.	Speech	85	15	Tender/Angry	Emotion	Ns	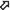
Kujala et al. (2007)	Kujala 2007a	10	8	30	27	EEG	Fz	Multi.	Tone	80	10	Complex tones	Frequency	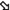	Ns
	Kujala 2007b										10	100/65 ms	Duration	Ns	Ns
	Kujala 2007c	10	8	30	27	EEG	Fz	Multi.	Tone	50	10	Complex tones	Frequency	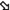	Ns
	Kujala 2007d										10	100/65 ms	Duration	Ns	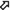
	Kujala 2007e										10	+ 10 ms	Gap	Ns	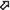
	Kujala 2007f										10	± 5 dB	Intensity	Ns	Ns
	Kujala 2007g										10	‐ 0.65 ms to the right or left ear	Location	Ns	Ns
Lepistö et al. (2007)	Lepisto 2007a	9	9	30	27	EEG	Fz	Multi.	Speech	76	8	113/125 Hz	Duration	Ns	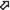
	Lepisto 2007b										8	190/104 ms	Frequency	Ns	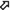
	Lepisto 2007c										8	≠ vowels	Phoneme	Ns	Ns
	Lepisto 2007d	9	9	30	27	EEG	Fz	Multi.	Tone	76	8	113/125 Hz	Duration	Ns	Ns
	Lepisto 2007e										8	190/104 ms	Frequency	Ns	Ns
	Lepisto 2007f										8	Tone/Vowel	Phoneme	Ns	Ns
Dunn et al. (2008)	Dunn 2008	34	34	9.5	9.2	EEG	Fz	Uni.	Tone	90	10	1000/1200 Hz	Frequency	Ns	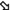
Kujala et al. (2010)	Kujala 2010a	13	15	10.5	10.8	EEG	Fz	Multi.	Speech	50	10	101/93 or 109 Hz	Frequency	Ns	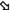
	Kujala 2010b										10	170/70 ms	Duration	Ns	Ns
	Kujala 2010c										10	55/49 or 61 dB	Intensity	Ns	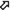
	Kujala 2010d										10	≠ consonant	Phoneme	NA	NA
	Kujala 2010e										10	≠ vowels	Phoneme	Ns	Ns
Gomot et al. (2011)	Gomot 2011	27	27	8.3	8.3	EEG	Fz	Uni.	Tone	85	15	1000/1100 Hz	Frequency	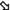	Ns
Roberts et al. (2011)	Not included	27	51	10.1	9.4	MEG	Bilateral STG source	Uni.	Tone	85	15	300/700 Hz	Frequency	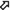	Ns
Andersson et al. (2013)	Andersson 2013	12	11	15.3	16	EEG	Fz	Uni.	Tone	90	10	75/25 ms	Duration	Ns	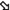
Ludlow et al. (2014)	Ludlow 2014a	11	11	13.7	13	EEG	Frontal	Multi.	Speech	80	10	≠ syllables	Phoneme	NA	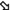
	Ludlow 2014b										10	≠ syllables	Phoneme	NA	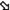
Abdeltawwab and Baz (2015)	Abdeltawwab 2015	30	31	11.2	11.3	EEG	Fz	Uni.	Tone	80	20	1100/1500 Hz	Frequency	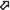	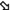
Weismüller et al. (2015)	Weismuller 2015a	15	18	10.6	9.4	EEG	Fz	Multi.	Tone	78.1	7.3	1000/1200 Hz	Frequency	Ns	Ns
	Weismuller 2015b										7.3	80/90 dB	Intensity	Ns	Ns
	Weismuller 2015c										7.3	50/100 ms	Duration	Ns	Ns
	Weismuller 2015d	15	18	10.6	9.4	EEG	Fz	Uni.	Speech	92	8	≠ syllables	Phoneme	Ns	Ns
Yu et al. (2015)	Yu 2015a	15	17	9.5	9.3	EEG	Fz	Uni.	Tone	84	16	216/299 Hz	Frequency	Ns	Ns
	Yu 2015b	16	18	9.6	9.3	EEG	Fz	Uni.	Speech	84	16	Word tonal change	Phoneme	Ns	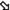
	Yu 2015c	16	18	9.6	9.3	EEG	Fz	Uni.	Speech	84	16	Pseudo‐word tonal change	Phoneme	Ns	Ns
Vlaskamp et al. (2017)	Vlaskamp 2017a	38	35	10.9	11.1	EEG	FCz	Multi.	Tone	82	6	1000/1200 Hz	Frequency	Ns	Ns
	Vlaskamp 2017b										6	50/100 ms	Duration	Ns	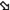
	Vlaskamp 2017c										6	1000/1200 Hz and 50/100 ms	Freq. & Dur.	Ns	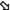
Chien et al. (2018)	Chien 2018a	35	37	20.5	21	EEG	Fz	Multi.	Tone	80	10	1000/1200 Hz	Frequency	Ns	Ns
	Chien 2018b										10	50/100 ms	Duration	Ns	Ns
Goris et al. (2018)	Goris 2018	24	18	NA (adults)		EEG	Fz	Uni.	Tone	80	20	Complex tone	Frequency	NA	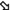
Hudac et al. (2018)	Hudac 2018	31	102	13.3	12.3	EEG	Central Medial	Uni.	Tone	70	15	1000/750 Hz	Frequency	Ns	Ns
Huang et al. (2018)	Huang 2018a	20	22	9.4	9.6	EEG	Cz	Uni.	Tone	84	16	250/350 ms	Duration	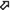	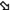
	Huang 2018b	17	18	9.4	9.8	EEG	Cz	Uni.	Speech	84	16	250/350 ms	Duration	Ns	Ns
Charpentier et al. (2018)	Not included	15	15	9.8	10	EEG	Frontal Central Parietal	Multi.	Speech	83	8.5	≠ vowels	Frequency	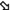	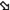
	Not included										8.5	Neutral/Angry	Emotion	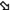	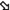
	Not included	16	16	26.2	26.2	EEG	Frontal Central Parietal	Multi.	Speech	83	8.5	≠ vowels	Frequency	Ns	Ns
	Not included										8.5	Neutral/Angry	Emotion	Ns	Ns
Lindström et al. (2018)	Lindstrom 2018a	16	15	10.1	10.4	EEG	Fz, Cz	Multi.	Speech	79	7	Neutral/Sad	Emotion	Ns	Ns
	Lindstrom 2018b										7	Neutral/Scornful	Emotion	Ns	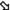
	Lindstrom 2018c										7	Neutral/Commanding	Emotion	Ns	Ns
Zhang et al. (2019)	Not included	16	16	9.5	10.4	EEG	Fz	Multi.	Speech	80	10	≠ pitch contour	Pitch contour	NA	Ns
	Not included										10	≠ pitch height	Pitch height	NA	NA
Di Lorenzo et al. (2020)	Di Lorenzo 2020a	20	21	14.2	14.3	EEG	Fz	Multi.	Tone	82.4	8.3	1000/1200 Hz	Frequency	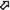	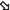
	Di Lorenzo 2020b										8.3	50/100 ms	Duration	Ns	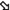
Okazaki et al. (2020)	Okazaki 2020	22	21	25.7	27	EEG	Fz	Uni.	Tone	80	20	1000/2000 Hz	Frequency	Ns	Ns
Ruiz‐Martínez et al. (2020)	Not included	15	16	8.9	9	EEG	F3, F4, Fz	Uni.	Tone	71.2	28.8	415/543, 494 or 554 Hz electronic tone	Frequency	Ns	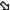
	Not included							Uni.	Tone	71.2	28.8	Singer mimicking the electronic tones	Frequency	Ns	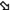
Riccioni et al. (2021)	Riccioni 2021	10	17	10.8	13.2	EEG	Fz	Uni.	Tone	85	15	Complex tones	Frequency	Ns	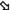
Randeniya et al. (2022)	Not included	23	23	24	24.4	EEG	Fz	Uni.	Tone	90	10	500/2000 Hz	Frequency	Ns	Ns
	Not included							Uni.	Tone	90	10	500/2000 Hz	Frequency	Ns	Ns
Leung et al. (2021)	Leung 2021	14	10	9.4	9.9	EEG	Cz, Fz, F4, F3	Multi.	Speech	70	10	Neutral/Angry, happy or sad	Emotion	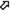	Ns
Lassen et al. (2022)	Lassen 2022a	59	59	11.8	11.9	EEG	Fz	Multi.	Tone	82	6	1000/1200 Hz	Frequency	Ns	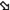
	Lassen 2022b										6	50/100 ms	Duration	Ns	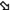
	Lassen 2022c										6	1000/1200 Hz and 50/100 ms	Freq. and Dur.	Ns	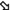
Cary et al. (2023)	Cary 2022	13	13	12.5	12.8	EEG	FCz	Uni.	Tone	80	20	1200/1000 Hz	Frequency	NA	Ns
Mayerle et al. (2023)	Mayerle 2023a	21	7	9.3	9.3	EEG	Fz	Uni.	Tone	90	10	1000/2000 Hz	Frequency	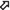	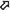
	Mayerle 2023b	30	10	13.6	13.6	EEG	Fz	Uni.	Tone	90	10	1000/2000 Hz	Frequency	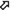	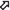
Matsuba et al. (2024)	Matsuba 2024	21	10	12.8	12.7	EEG	Fronto‐central	Uni.	Speech	90	10	≠ syllables	Phoneme	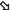	Ns
Schall et al. (2024)	Schall 2024a	13	17	7.9		EEG	Fz	Uni.	Speech	92	8	≠ syllables	Phoneme	NA	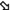
	Schall 2024b	17	18	9.7		EEG	Fz	Uni.	Speech	92	8	≠ syllables	Phoneme	NA	Ns
	Schall 2024c	17	18	10.4		EEG	Fz	Uni.	Speech	92	8	≠ syllables	Phoneme	NA	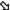
	Schall 2024d	17	12	11.6		EEG	Fz	Uni.	Speech	92	8	≠ syllables	Phoneme	NA	Ns
	Schall 2024e	15	13	7.9		EEG	Fz	Multi.	Tone	85.4	7.3	50/100 ms	Duration	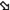	Ns
	Not included										7.3	1000/1200 Hz	Frequency	Ns	Ns
	Schall 2024f	14	17	8.5		EEG	Fz	Multi.	Tone	85.4	7.3	50/100 ms	Duration	Ns	Ns
	Not included										7.3	1000/1200 Hz	Frequency	Ns	Ns
	Schall 2024g	13	17	9.6		EEG	Fz	Multi.	Tone	85.4	7.3	50/100 ms	Duration	Ns	Ns
	Not included										7.3	1000/1200 Hz	Frequency	Ns	Ns

*Note*: The studies included in the meta‐analysis of MMN amplitude and/or latency are specified in the second column. The column entitled “MMN in ASD” indicates whether MMN was significantly increased (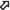) or decreased (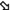) in amplitude and latency in the ASD group, compared to the NT group.

Abbreviations: ASD, Autism Spectrum Disorders; Freq. and Dur, deviant in both frequency and duration; *N*, number of participants; NA, data not available; Ns, no significant group difference; NT, Neurotypical; P(dvt), probability of the deviant stimulus; P(std), probability of the standard stimulus; Uni./Multi., Unifeature/Multifeature.

Additionally, studies were excluded from the meta‐analysis if they: (i) did not report the mean MMN response for either amplitude or latency (note that studies were retained if at least one of these measures was reported), (ii) used a roving paradigm (which was uncommon as the majority of studies employed oddball paradigms), or (iii) used MEG instead of EEG (to ensure methodological consistency for Hedges' g calculation). These additional criteria were applied to improve comparability across studies and to allow for the calculation of Hedges' g in amplitude and/or latency. Studies excluded based on these criteria are marked as “*Not included”*, but are reported in Table 1 for review purposes.

To ensure accuracy, data were extracted independently twice and cross‐checked for potential errors. The extracted data were also compared with findings from two previous MMN meta‐analyses on ASD (Schwartz et al. 2018; Chen et al. 2020), which included studies published up to September 2018. No protocol was registered for this meta‐analysis. The PRISMA checklist is provided as Table S1.

### 2.2 Data Extraction and Synthesis

For each article, the following data regarding the experimental setup were extracted: number of participants, groups (i.e., ASD or NT), mean age, recording technique (i.e., EEG or MEG), electrode(s) used to extract the MMN, experimental design (i.e., *unifeature* if only one type of deviant was used, or *multifeature* if two or more types of deviants were included in each block), stimulus type (i.e., *tone* or *speech*), probabilities of deviant and standard stimuli, the features of the standard and deviant tones (e.g., standard at 1000 Hz and deviant at 1200 Hz), and the type of deviant stimuli (e.g., differing in *frequency*, *phoneme*, *duration*, both *frequency and duration*, *emotion*, *intensity*, or *gap*). These categories of deviant types are detailed in Table 1, however, for the analyses, they were grouped into four conditions: *frequency*, *phoneme*, *duration*, and *other*. The *other* category encompassed deviants in both *frequency and duration*, or in *emotion*, *intensity*, and *gap* (i.e., length of the interstimulus interval), as these represented a smaller subset of studies. Furthermore, studies were categorized into two age groups: *children and adolescents* for participants under 18 years old (age range across studies: 5–17 years), and *adults* for participants above 18 years old (age range across studies: 18–50 years). Additional population characteristics (i.e., diagnostic status, cognitive assessments) are provided in Table S2.

Additionally, the following results were extracted: the mean and standard deviation (or variance) of the MMN amplitude and latency for each group, and whether a significant group difference was observed in MMN amplitude or latency. For studies that distinguished between early and late MMN responses, we extracted data for the MMN within the 100–400 ms range, which aligned with the time window reported in studies providing a single MMN response.

As in previous MMN meta‐analyses (Chen et al. 2020), if an article reported multiple tasks or employed a multifeature design (e.g., 80% standard stimuli, 10% frequency deviants, 10% duration deviants), each condition or task was treated as a separate study. However, information regarding whether the design was multifeature or unifeature was taken into account in the analyses, and multivariate analyses were conducted to account for the dependency of the measures in multifeature paradigms.

### 2.3 Meta‐Analysis

In this manuscript, all the results are presented as Mean (± Standard Deviation). The threshold for statistical significance was set at *p* < 0.05. Statistical analyses were performed using R (version 4.3.2, http://www.r‐project.org/).

#### 2.3.1 Effect Size

For each study included in the meta‐analysis, we computed the effect size of the group difference as Hedges' *g*, as well as the 95% confidence interval (CI). Based on the mean (M) and standard deviation (SD) of the MMN, in amplitude or latency, in the NT and ASD groups, Hedges' *g* was calculated as:

$${\text{Hedges}}^{’}\;g=\frac{M_{\mathrm{NT}}-M_{\mathrm{ASD}}}{\sqrt{\frac{{\mathrm{SD}}_{\mathrm{NT}}^{2}+{\mathrm{SD}}_{\mathrm{ASD}}^{2}}{2}}}$$

Note that for studies reporting the standard error of the mean (SEM) instead of the SD, the SD was calculated using the formula $\mathrm{SD}=\mathrm{SEM}*\sqrt{N}$, where N is the number of participants.

The weights assigned to each effect size are a function of the sample size (*N*_NT_ and *N*_ASD_) and effect size magnitude (Hedges' *g*), ensuring that studies with small sample sizes do not exert disproportionate influence on the results. The variance of Hedges' g values (SE) was calculated as follows:

$$\mathrm{SE}=\sqrt{\frac{N_{\mathrm{NT}}+N_{\mathrm{ASD}}}{N_{\mathrm{NT}}\times N_{\mathrm{ASD}}}+\frac{g²}{2\times \left(N_{\mathrm{NT}}+N_{\mathrm{ASD}}\right)}}$$

#### 2.3.2 Multivariate Random‐Effects Meta‐Analyses

To assess effect sizes across studies, we conducted multivariate random‐effects meta‐analyses, using the *rma.mv* function from the metafor package in R. First, multivariate meta‐analyses were performed on the entire dataset for both Hedges' g MMN amplitude and latency measures. The restricted maximum‐likelihood estimation method was used to estimate between‐study variance and calculate the summary effect size for each measure. In the model structure, random effects were specified at two levels: (1) between studies and (2) within studies, nested by MMN deviant type, to account for dependency structures in multifeature designs where multiple deviant types were included per study.

Next, additional multivariate meta‐analyses were conducted to examine the contributions of various study‐level characteristics to the observed heterogeneity in effect sizes. These characteristics included two continuous and three categorical moderators. The continuous moderators were the probabilities of standard and deviant stimuli. The categorical moderators included age group (*Children/Adolescents* vs. *Adults*, with *Adults* as the reference group), MMN deviant condition (*Frequency*, *Duration*, *Phoneme*, *Other*, with *Frequency* as the reference group), and design type (*Unifeature* vs. *Multifeature*, with *Unifeature* as the reference group). Interaction terms were tested between age group and design type to evaluate whether the relationships between group differences and design depended on age category. These additional meta‐analyses helped interpret the influence of these moderators on MMN effect sizes and to visualize the data accordingly. The degrees of heterogeneity were assessed using Q‐statistics and the significance of moderators using QM‐tests (Alexander et al. 1989). When moderator or interaction effects were identified, stratified subgroup meta‐analyses were conducted.

#### 2.3.3 Publication Bias Assessment

To evaluate publication bias, we fitted univariate random‐effects models (one effect size per study) to allow standard diagnostic procedures (e.g., funnel plot and Egger's test), which are not available for multivariate models. Funnel plots were presented to illustrate potential publication biases. Egger's tests were used to assess funnel plot asymmetry and evaluate publication biases. To investigate whether any single study heavily influenced the overall meta‐analyses, leave‐one‐out analyses were conducted.

## 3 Results

### 3.1 Descriptive Statistics of Studies Included in the Literature Review

Our systematic literature review identified 38 articles that met the eligibility criteria (Figure 1, Table 1), encompassing 97 studies. Most studies (*n* = 72) were conducted with children; the median age was 10.6 years, and studies had relatively low sample sizes (median sample size of 15 participants per group). Most studies relied on multifeature designs rather than unifeature designs (63% vs. 37%), with a median deviant probability of 10%. The majority of studies used deviants in frequency (40%), duration (23%), or phoneme (21%), while the remainder investigated other types of deviants (e.g., emotion, intensity). Additionally, most studies used tone stimuli (60%) rather than speech stimuli (40%).

Regarding MMN amplitude, 60% of studies reported no significant group differences, while 28% reported significantly smaller MMN amplitudes in ASD (i.e., more positive MMN), and 10% reported significantly larger MMN amplitudes in ASD (i.e., more negative MMN). The remaining 2% of studies did not report or compare MMN amplitudes.

Regarding MMN latency, 66% of studies reported no significant group differences, while 9% reported significantly shorter MMN latencies in ASD, and 13% reported significantly longer MMN latencies in ASD. The remaining 12% of studies did not report or compare MMN latencies.

These 38 articles are summarized in Table 1 for review purposes. However, the meta‐analysis reported below included only 32 articles, as it focused exclusively on studies that reported MMN amplitude or latency values using EEG, as such data are necessary to carry out meta‐analyses across articles (see Section 2).

### 3.2 Descriptive Statistics of Studies Included in the Meta‐Analysis

#### 3.2.1 MMN Amplitude

Thirty articles reported MMN amplitudes and included a total of 76 studies, as several articles included multiple studies or deviant types per experiment (Figure 1D–F). Sixty‐four percent of the studies used multifeature designs (vs. 36% using unifeature designs), with an average deviant probability of 10%. Deviant types included frequency (36%), duration (25%), phoneme (25%), and other features (14%), with stimuli being tones (59%) or speech (41%). Twenty‐two percent of the studies involved adults (27.4 ± 2.6 years, 13.6 ± 9.3 participants per group) and 78% involved children or adolescents (10.3 ± 1.8 years, 17.3 ± 10.5 participants per group).

The average MMN amplitude was −3.5 μV (± 1.7) in children and adolescents and −2.5 μV (± 1.0) in adults. Note that these averages exclude seven studies that reported the difference in MMN amplitude between NT and ASD groups but did not provide the average amplitude for each group.

**FIGURE 2:**
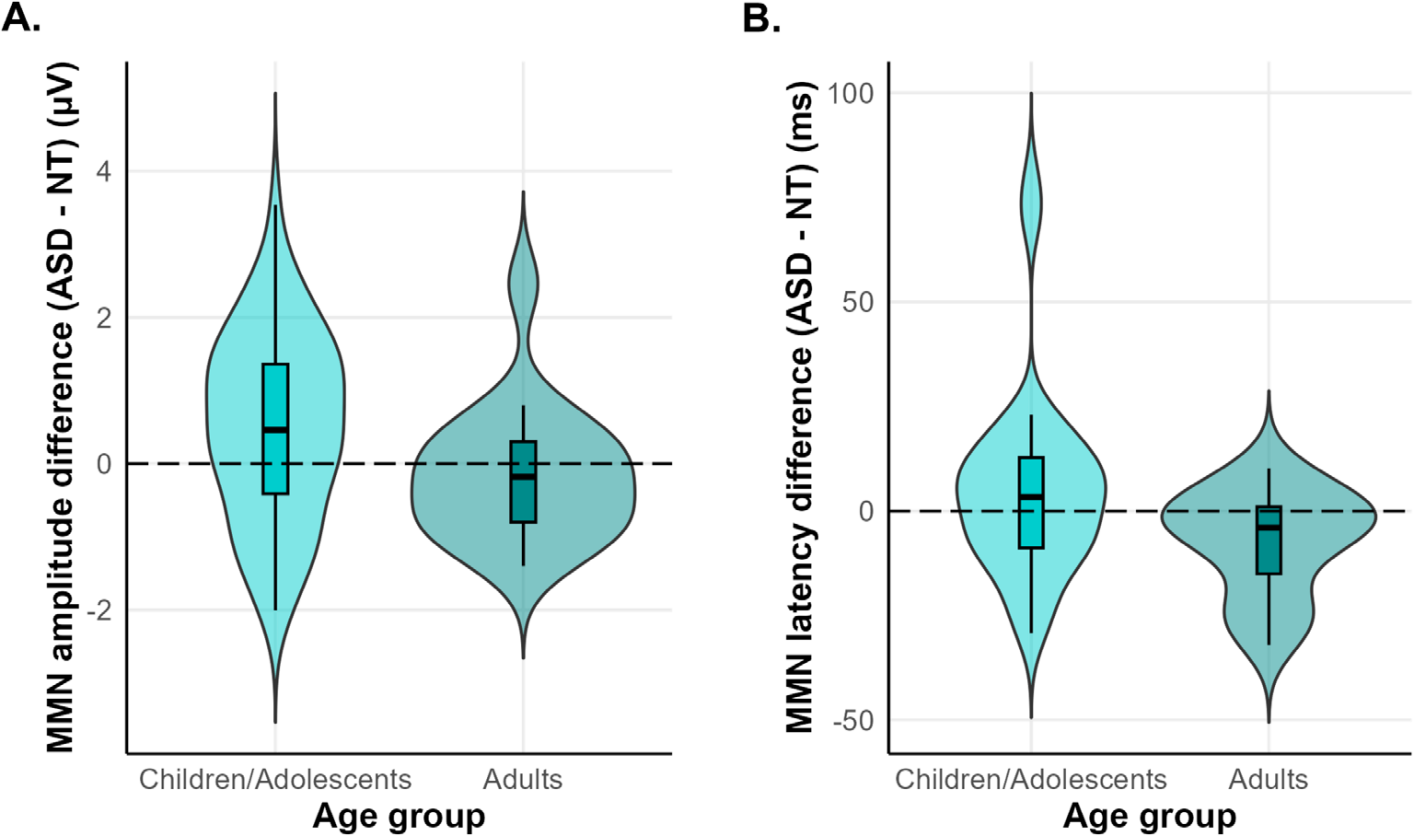
Group differences in MMN amplitude (A) and latency (B) between ASD and NT groups across the studies included in the meta‐analysis. (A) Positive values indicate smaller (less negative) MMN amplitudes in the ASD group compared to the NT group. Negative values indicate larger (more negative) MMN amplitudes in the ASD group. (B) Positive values indicate a delayed MMN peaks in the ASD group compared to the NT group. Negative values indicate earlier MMN peaks in the ASD group.

Group differences in MMN amplitudes are illustrated in Figure 2A. Among children and adolescents, the MMN amplitude was 0.5 μV (± 1.3) smaller in the ASD group than in the NT group. Among adults, it was 0.2 μV (± 0.9) larger in the ASD group than in the NT group.

#### 3.2.2 MMN Latency

Twenty‐six articles reported MMN latencies, corresponding to 62 studies (Figure 1E–G). Of these studies, 68% used multifeature designs (vs. 32% using unifeature designs), with an average deviant probability of 10%. Deviant types included frequency (39%), duration (29%), phoneme (18%), and other features (15%), with stimuli being tones (65%) or speech (35%). Twenty‐six percent involved adults (27.4 ± 2.6 years, 13.2 ± 9.3 participants per group) and 74% involved children or adolescents (10.2 ± 1.9 years, 17.7 ± 11.4 participants per group).

The average MMN latency was 222.7 ms (± 90.0) in children and adolescents and 165.6 ms (± 40.3) in adults. Note that these averages exclude three studies that reported the difference in MMN latency between NT and ASD participants but did not provide the average latency for each group.

Group differences in MMN latencies are illustrated in Figure 2B. Among children and adolescents, MMN latencies were 5.1 ms longer (± 22.7) in the ASD group than in the NT group. Among adults, they were 7.4 ms shorter (± 12.6) in the ASD group than in the NT group.

### 3.3 Meta‐Analytic Results on MMN Amplitude

#### 3.3.1 MMN Amplitude: Multivariate Meta‐Analysis Without Moderators

**FIGURE 3:**
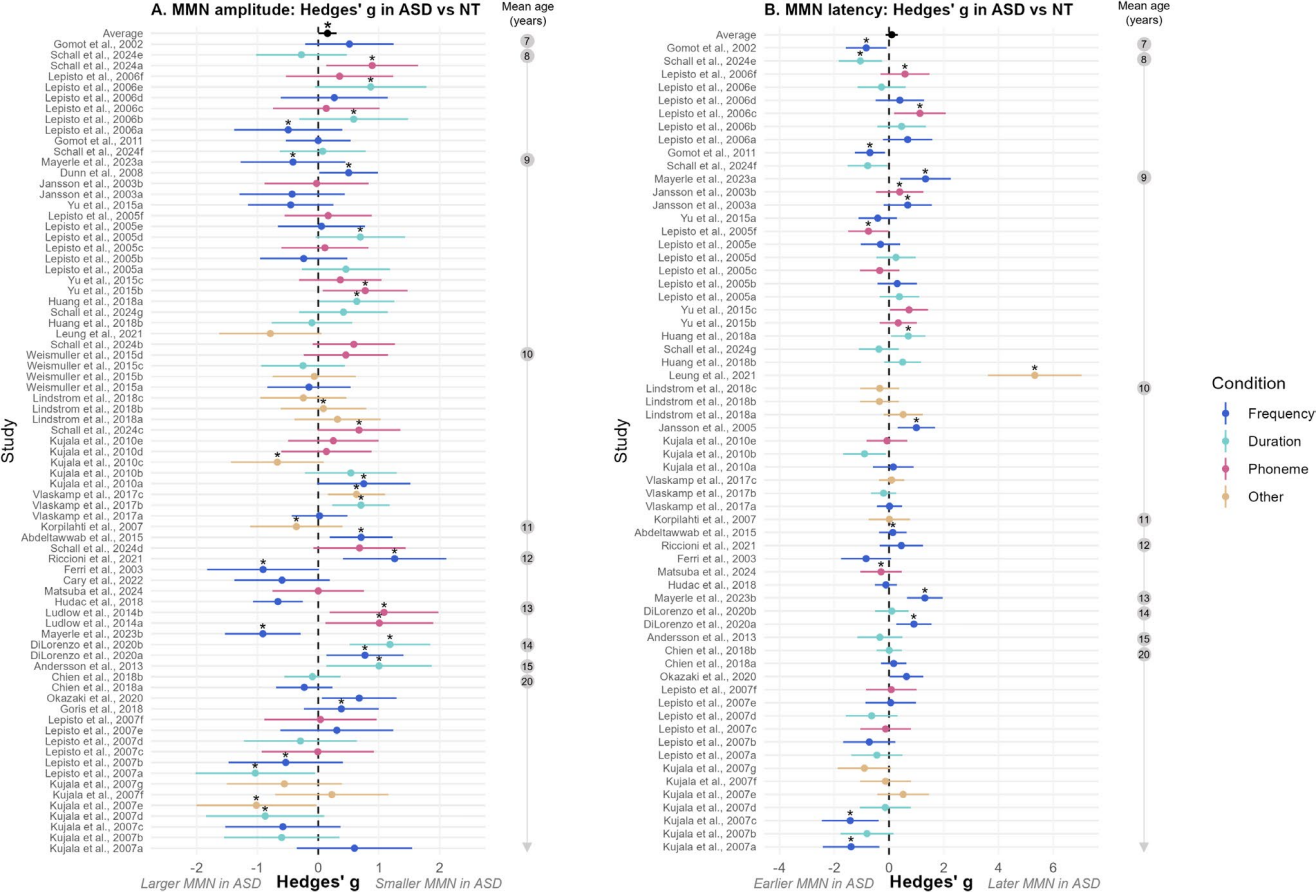
Forest plots showing group differences in MMN amplitude (A) and latency (B) between ASD and NT groups. Each data point represents the effect size (Hedges' *g*) for MMN amplitude or latency differences between ASD and NT groups, with error bars representing 95% confidence intervals. Stars above the data points indicate articles that reported significant group differences for the corresponding variable. Data points are color‐coded based on the type of deviant: frequency (dark blue), duration (light blue), phoneme (pink), or other features (e.g., emotion, gap, intensity, location, or combined duration and frequency; light brown). Studies were ordered by mean age (younger groups in the upper part, older groups in the lower part of the plot). For the overall effect size, the error bars around the average data point (black) reflect the multivariate meta‐analytical confidence intervals. (A) On average, MMN amplitude differed significantly between ASD and NT groups. (B) On average, MMN latency did not differ significantly between ASD and NT groups.

A multivariate random‐effects meta‐analysis was conducted to assess Hedges' *g* MMN amplitude values across the 76 measures (Figure 3A), while appropriately accounting for statistical dependencies within studies that reported multiple deviant types within the same block (i.e., multifeature designs). The overall effect size was 0.15 (95% CI [0.00, 0.30]) and was statistically significant (*p* = 0.046). This result indicates a small but significant difference in MMN amplitude between NT and ASD individuals.

There was residual heterogeneity in the data (*Q* = 168.5, *p* < 0.001), suggesting substantial variability in effect sizes across studies that may be explained by specific study‐level or methodological characteristics. Therefore, we extended the multivariate meta‐analysis model to evaluate the effect of potential moderators.

#### 3.3.2 MMN Amplitude: Multivariate Meta‐Analysis With Moderators

A multivariate meta‐analysis was conducted to evaluate the influence of age group (children and adolescents vs. adults), design type (unifeature vs. multifeature), their interaction, standard and deviant probabilities, and deviant type on MMN amplitude effect sizes. The model showed that moderators significantly explained variability in effect sizes (QM = 21.4, *p* = 0.006). After accounting for moderators, residual heterogeneity remained (QE = 118.0, *p* < 0.001). Two significant predictors of group differences in MMN amplitude effect sizes were identified: the interaction between age category and design type and the deviant type.

The interaction between age category and design type was a significant moderator of MMN amplitude effect sizes (*β* = 0.93, 95% CI [0.11, 1.74], *p* = 0.027), indicating that the relationship between design and MMN group differences varied by age group.

In addition, the type of deviant stimulus significantly influenced MMN amplitude differences: studies using phoneme deviants yielded significantly larger group differences than frequency deviants (*β* = 0.38, 95% CI [0.08, 0.68], *p* = 0.013).

The probabilities of standard stimuli showed a non‐significant trend toward predicting effect sizes (*β* = 0.015, 95% CI [0.00, 0.03], *p* = 0.085).

Based on these predictors, further analyses were conducted by splitting the data on MMN amplitude according to both age group (children/adolescents vs. adults) and design type (unifeature vs. multifeature), as well as deviant type.

#### 3.3.3 MMN Amplitude: Post Hoc Analyses

##### 3.3.3.1 Subgroup Analyses by Age Group and Design

###### 3.3.3.1.1 Unifeature Designs

In the subset of 25 studies using unifeature designs with children or adolescents, the overall effect size was *g* = 0.17 (95% CI [−0.07, 0.41]) and was not statistically significant (*p* = 0.17). There was substantial heterogeneity across studies (*Q* = 77.4, *p* < 0.0001). In sum, autistic and NT children did not significantly differ in MMN amplitude in unifeature designs.

As only two studies with adult participants using unifeature designs reported MMN amplitudes, no analyses were conducted. Both studies had positive Hedges' g values (i.e., in favor of smaller MMN responses in ASD).

###### 3.3.3.1.2 Multifeature Designs

**FIGURE 4:**
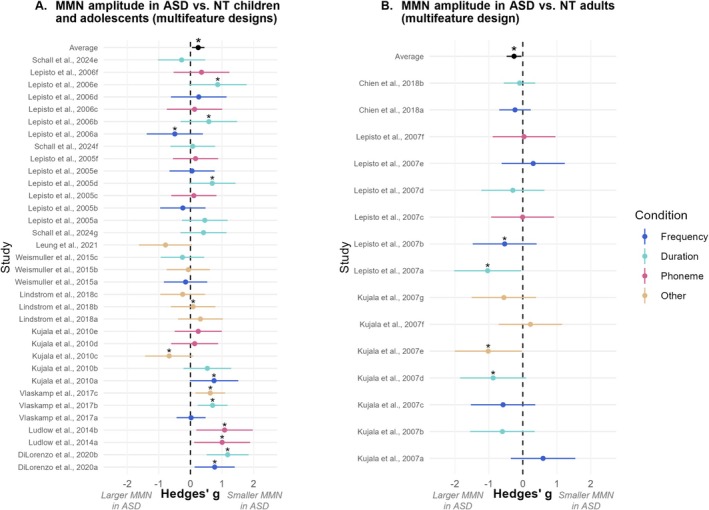
Forest plots showing MMN amplitude differences between ASD and NT groups in multifeature designs. Each data point represents the effect sizes (Hedges' *g*) for MMN amplitude differences between the ASD and NT groups, with error bars representing 95% confidence intervals. Stars above the data points indicate studies that reported a significant group difference for this variable. These data points were colored according to the type of deviant: Frequency (dark blue), duration (light blue), phoneme (pink), or other features (light brown). Studies were ordered by mean age. Average data point: *p < 0.05. (A) In multifeature designs, autistic children and adolescents had smaller MMN amplitudes (i.e., less negative MMN values) than typically developing peers. (B) In multifeature designs, autistic adults had larger MMN amplitudes (i.e., more negative MMN values) than neurotypical adults.

In the subset of 34 studies using multifeature designs with children and adolescents, the overall effect size was *g* = 0.25 (95% CI [0.05, 0.44]) and was statistically significant (*p* = 0.01). There was moderate heterogeneity across studies (*Q* = 54.5, *p* = 0.01). These results indicate that autistic children and adolescents had significantly smaller MMN amplitudes than their NT peers in multifeature designs (Figure 4A).

In the subset of 15 studies using multifeature designs with adults, the overall effect size was *g* = −0.26 (95% CI [−0.48, −0.04]) and was statistically significant (*p* = 0.02). There was no significant heterogeneity across studies (*Q* = 14.6, *p* = 0.41). Results show that autistic adults had significantly larger MMN amplitudes (i.e., more negative MMN responses) than NT adults in multifeature designs (Figure 4B).

##### 3.3.3.2 Subgroup Analyses by Deviant Type

###### 3.3.3.2.1 Frequency Deviants

**FIGURE 5:**
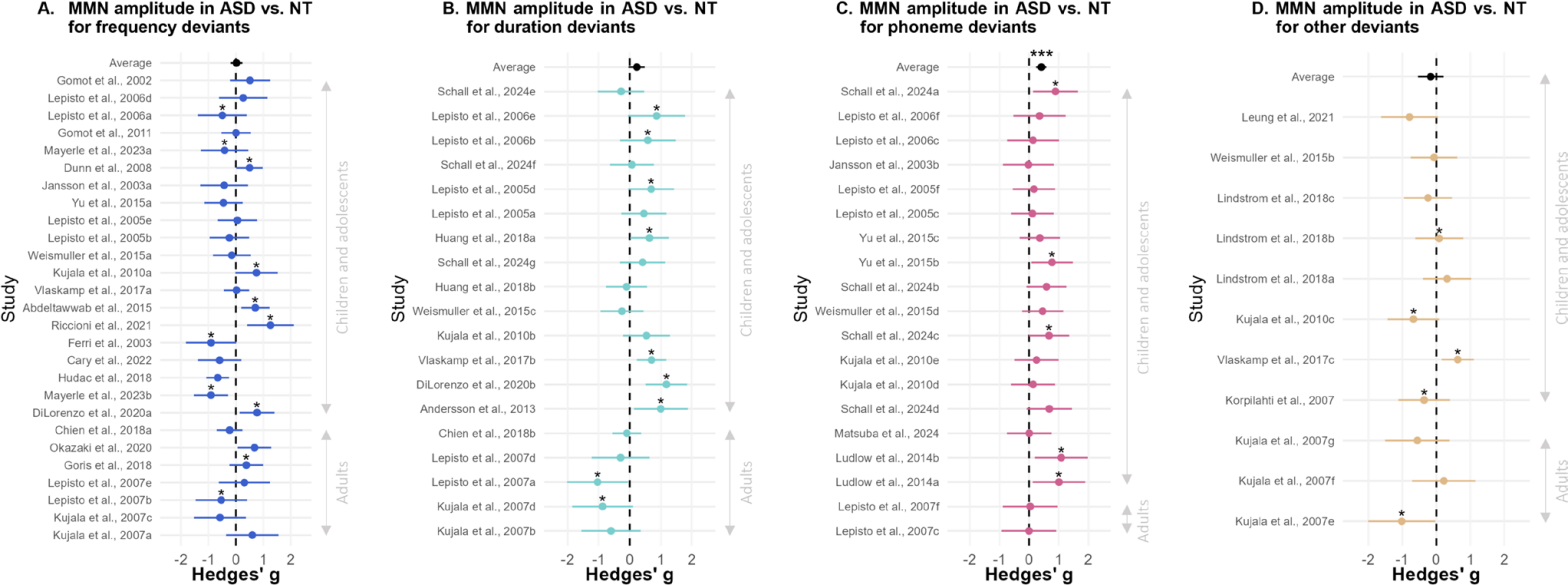
Forest plots showing MMN amplitude differences between ASD and NT groups for each deviant type. Each data point represents the effect sizes (Hedges' *g*) for MMN amplitude group differences, with error bars representing 95% confidence intervals. Stars above data points indicate studies that reported a significant group difference for this variable. Studies were ordered by mean age. Average datapoint: ***p < 0.001. In designs using deviants in frequency (A), duration (B) or other (D), no significant differences in MMN amplitude were found between NT and ASD groups. In designs using deviants in phoneme (C), MMN amplitude was significantly smaller in ASD than in NT individuals.

In the subset of 27 studies using deviants in frequency, the overall effect size was *g* = 0.02 (95% CI [−0.20, 0.24]) and was not statistically significant (*p* = 0.87). There was substantial heterogeneity across studies (*Q* = 72.4, *p* < 0.0001). In sum, ASD and NT individuals did not differ in MMN amplitude for frequency deviants (Figure 5A).

###### 3.3.3.2.2 Duration Deviants

In the subset of 19 studies using deviants in duration, the overall effect size was *g* = 0.23 (95% CI [−0.04, 0.49]) and was not statistically significant (*p* = 0.09). There was moderate heterogeneity across studies (*Q* = 43.6, *p* < 0.001). Although there was a non‐significant trend toward decreased MMN amplitude in ASD, no significant difference was observed for duration deviants (Figure 5B).

###### 3.3.3.2.3 Phoneme Deviants

In the subset of 19 studies using deviants in phoneme, the overall effect size was *g* = 0.41 (95% CI [0.24, 0.59]) and was statistically significant (*p* < 0.001). There was no significant heterogeneity across studies (*Q* = 13.6, *p* = 0.75). These results indicate that autistic individuals had significantly smaller MMN amplitudes than NT individuals in paradigms using phoneme deviant stimuli (Figure 5C).

###### 3.3.3.2.4 Other Deviants

In the subset of 11 studies using other types of deviants, the overall effect size was *g* = −0.17 (95% CI [−0.54, 0.21]), which was not statistically significant (*p* = 0.38). There was significant heterogeneity across studies (*Q* = 20.7, *p* = 0.02). In these other designs, there was no evidence of statistical differences in MMN amplitude between groups (Figure 5D).

### 3.4 Meta‐Analytic Results on MMN Latency

#### 3.4.1 MMN Latency: Multivariate Meta‐Analysis Without Moderators

A multivariate random‐effects meta‐analysis was conducted to assess Hedges' *g* for MMN latency across the 62 studies (Figure 3B). The overall effect size estimate was 0.10 (95% CI [−0.12, 0.33]) and was not statistically significant (*p* = 0.38). This indicates that there is no evidence of a difference in MMN latency between NT and ASD individuals across the included studies.

There was residual heterogeneity in the data (*Q* = 182.1, *p* < 0.0001), suggesting substantial variability in effect sizes across the studies. Therefore, we extended the multivariate meta‐analysis model to evaluate the effect of potential moderators.

#### 3.4.2 MMN Latency: Multivariate Meta‐Analysis With Moderators

A multivariate meta‐analysis was conducted to assess whether MMN latency differences between ASD and NT individuals were moderated by age group, design type, their interaction, deviant type, and the probabilities of standard and deviant stimuli. After accounting for the moderators, substantial heterogeneity remained (QE = 163.1, *p* < 0.0001). The overall test of moderators was not significant (QM = 7.9, *p* = 0.44), indicating that these moderators explained only a minimal portion of the variability in effect sizes across studies. Therefore, no additional analyses were performed to examine MMN latency separately by moderator.

### 3.5 Publication Bias

**FIGURE 6:**
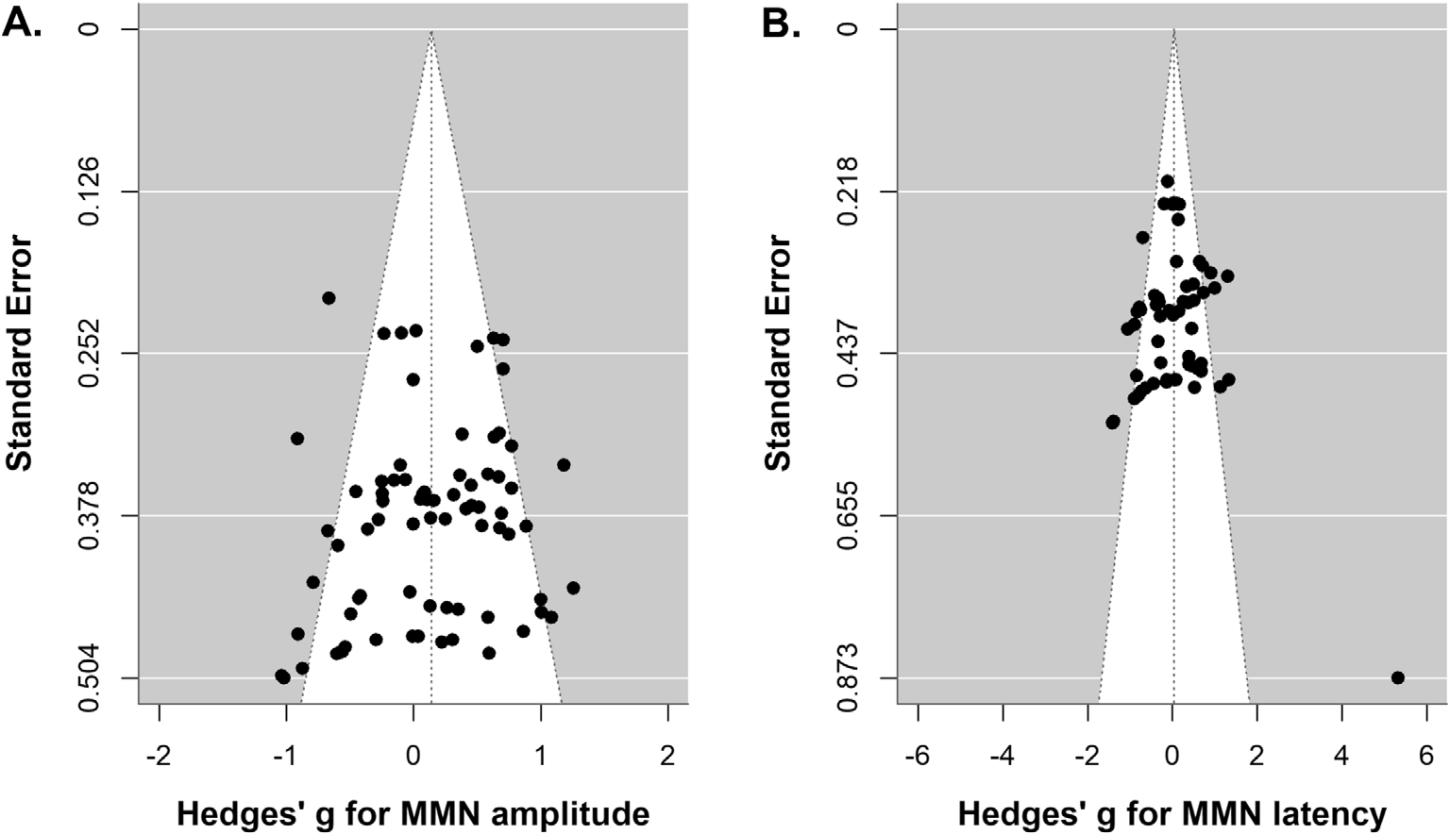
Funnel plots for MMN amplitude (A) and latency (B). Symmetrical funnel plots suggest no evidence of publication bias. Each data point represents the effect sizes (Hedges' *g*) for MMN amplitude (A) or latency (B) differences between ASD and NT groups in studies included in the meta‐analysis.

Funnel plots are presented in Figure 6. Egger's test indicated non‐significant funnel plot asymmetry for both MMN amplitude (*z* = −1.5, *p* = 0.13) and latency (*z* = 1.2, *p* = 0.25), suggesting no evidence of publication bias or small‐study effects. The intercept estimates for MMN amplitude (*b* = 0.60, 95% CI [−0.01, 1.21]) and latency (*b* = −0.37, 95% CI [−1.10, 0.35]) suggest no evidence that smaller studies report larger effect sizes.

To further investigate how much each study influenced the overall meta‐analytic results, leave‐one‐out analyses were conducted. For MMN amplitudes, the leave‐one‐out analyses showed that the pooled effect size estimates remained consistent when individual studies were excluded (values ranging between 0.12 and 0.15) and heterogeneity showed minimal changes (remaining around 55%). For MMN latencies, the pooled effect size estimates also remained stable (values ranging between 0.01 and 0.06) and heterogeneity showed minimal changes (remaining around 68%). These results suggest that no single study had an undue influence on the overall findings of the meta‐analysis on MMN.

## 4 Discussion

This meta‐analysis examined the auditory MMN response in autistic individuals to identify factors contributing to heterogeneity in MMN findings and to interpret results within the predictive coding framework. Overall, MMN amplitude differed between NT and ASD individuals, whereas MMN latency did not differ. Several moderators explained variability in effect sizes across studies for MMN amplitudes, revealing group differences when studies were subset according to these moderators. Specifically, an interaction between age group and study design, as well as deviant type, influenced group differences in MMN amplitude. In multifeature designs, autistic children and adolescents showed a significantly smaller MMN compared to their NT peers, whereas autistic adults exhibited a significantly larger MMN than NT adults. For MMN latency, no moderators significantly accounted for variability in effect sizes.

### 4.1 Group Differences in MMN Amplitude in Multifeature Designs

While the meta‐analysis showed no significant group difference in MMN latency, differences in MMN amplitude were observed. Indeed, group differences in MMN amplitude between ASD and NT individuals were found in multifeature designs, but in opposite directions depending on age group. Reduced MMN amplitude in autistic children may reflect atypical early detection of deviance, pre‐attentional abnormalities in auditory discrimination (Hudac et al. 2018), or poor automatic discrimination of irregularities in the absence of attention (Dunn et al. 2008). In contrast, in autistic adults, an increased MMN amplitude has been interpreted as reduced neural adaptation to repeated sensory inputs, difficulties in updating expectations to sequences of stimuli, or temporal cortex dysfunction influencing pre‐perceptual auditory processing (Ferri et al. 2003). From a predictive coding perspective, decreased MMN amplitude in autistic children may be interpreted as weaker adaptation to standard stimuli in uncertain contexts, leading to unclear distinctions between standard and deviant stimuli. Conversely, increased MMN amplitude in autistic adults may reflect enhanced discrimination between standard and deviant stimuli, and/or greater precision of prediction errors. This hypothesis aligns with the theory of an inflexibly high weighting of prediction errors in ASD (HIPPEA hypothesis) (Van de Cruys et al. 2014). Importantly, these interpretations remain speculative and should be tested using MMN paradigms combined with computational modeling designed to disentangle these hypotheses. Overweighting prediction errors should, theoretically, lead to faster prior updates. This is in line with an MMN study that suggested faster model updating during early sensory processing in ASD, as primacy bias in MMN amplitude was observed in NT but not in ASD adults (Goris et al. 2022). Primacy bias refers to the high influence of initial stimuli in an oddball sequence on later deviance detection, illustrating the persistent impact of early learning on MMN (Mullens et al. 2014). Yet, alternatively, the decreased primacy bias in ASD observed in their study could reflect slower prior learning, as the initial blocks may have been too short to establish a prior, preventing its influence on subsequent blocks. Additionally, another MMN study suggested inflexible precision of prediction errors in ASD (Goris et al. 2018), as autistic adults showed reduced context‐dependent modulation of MMN amplitude.

The opposite patterns in MMN amplitude observed in multifeature designs depending on age groups (i.e., reduced MMN amplitude in children/adolescents and increased amplitude in adults) suggest underlying developmental changes. These findings raise questions about how brain maturation impacts MMN response in ASD and NT individuals, particularly given that ASD is a neurodevelopmental disorder. A study using the same oddball paradigm across different age groups found that young autistic children (6–8 years old) lacked an observable MMN, which gradually emerged in older age groups (Dunn et al. 2008). Hence, decreased MMN amplitude in the children/adolescent group may partly reflect younger autistic children who lack MMN responses. Moreover, developmental changes in NT individuals may have accentuated group differences in MMN amplitude in autistic adults. Indeed, across typical development, MMN responses not only change in topographic distribution (Martin et al. 2003), but also show a decrease in amplitude (Czigler et al. 1992). This decrease in MMN amplitude from childhood to adulthood was also observed in the present meta‐analysis. If MMN amplitude decreases more in typical development than in atypical development, this may contribute to the seemingly increased MMN amplitude in autistic adults. To precisely characterize MMN changes in ASD across the lifespan, future studies should consider treating age as a continuous variable, rather than dividing it into broad age groups. In this meta‐analysis, age was treated categorically because many studies included participants spanning broad age ranges and a few did not report mean ages. Longitudinal studies are needed to investigate how and why MMN response patterns change during development in ASD compared to NT. Notably, predictive coding theories do not explicitly account for developmental changes in predictive processes in ASD. Based on this meta‐analysis, one hypothesis is that statistical regularities are harder to learn in autistic children under uncertain conditions, while this ability improves in adulthood and is accompanied by an enhanced weighting of prediction errors.

These results also indicate that simple, unifeature oddball paradigms are less likely to distinguish ASD from NT individuals. Introducing more subtle paradigm variations, such as manipulating the dynamics of standard and deviant stimulus probabilities and using multifeature designs, may better differentiate these groups. In addition, MMN reflects only the average response to deviant stimuli compared to preceding standard stimuli and does not capture learning dynamics. In contrast, in other experimental contexts, group differences often emerge only when such dynamics are modeled (Sapey‐Triomphe, Weilnhammer, and Wagemans 2021; Lieder et al. 2019; Lawson et al. 2017; Sapey‐Triomphe, Sanchez, et al. 2023). Temporally decomposing MMN can help uncover these mechanisms, as multiple processes may contribute at different latencies. For instance, early MMN components are better explained by adaptation mechanisms, while later components align more closely with Bayesian learning models (Lecaignard et al. 2022; Poublan‐Couzardot et al. 2023). It is therefore important to consider trial‐by‐trial fluctuations rather than relying solely on averaged MMN responses to fully capture the learning dynamics.

### 4.2 Group Differences in MMN Amplitude According to Deviant Type

Autistic individuals showed decreased MMN amplitudes in response to phoneme deviants. Notably, 17 out of the 19 studies included in this analysis involved children or adolescents, suggesting that this result mostly reflects effects observed in younger populations. This result is consistent with a previous meta‐analysis (Chen et al. 2020) reporting reduced MMN amplitudes to speech‐sound deviants in autistic individuals. While their meta‐analysis (Chen et al. 2020) also found increased MMN latencies to such deviants, our own analysis did not reveal significant moderating effects for MMN latency. Taken together, their findings (Chen et al. 2020) and ours suggest that autistic individuals may have difficulties in differentiating discrete units of speech sounds. This automatic neural discrimination of speech sounds is essential for phonological processing and language acquisition and may therefore contribute to language learning difficulties and broader communication impairments in ASD (American Psychiatric Association 2013). In contrast, an earlier meta‐analysis (Schwartz et al. 2018) reported decreased MMN amplitudes in young autistic children specifically for non‐speech sounds, but not for speech sounds.

In addition to the decreased MMN amplitude in response to speech deviants, the previous meta‐analysis (Chen et al. 2020) also reported reduced MMN amplitudes in response to duration deviants in autistic children and adolescents. Although this effect was not statistically significant in our meta‐analysis, we observed a similar trend toward decreased MMN amplitudes for duration deviants, probably driven by studies involving children and adolescents. This finding may reflect challenges in temporal auditory processing in ASD, which may contribute to reduced performance on temporal reproduction tasks in autistic children (Karaminis et al. 2016; Maister and Plaisted‐Grant 2011).

Finally, consistent with prior meta‐analyses (Schwartz et al. 2018; Chen et al. 2020), the present study did not reveal significant group differences in response to frequency deviants, suggesting intact processing of such deviants.

### 4.3 Links Between MMN and ASD Symptoms

Several studies have attempted to link MMN responses to ASD symptoms or behavioral characteristics, but most have failed to demonstrate a clear association. In ASD, no relationships were found between MMN responses and nonverbal cognitive abilities (Hudac et al. 2018), language abilities (Dunn et al. 2008; Ferri et al. 2003), autistic traits, or behavioral rigidity (Lassen et al. 2022). However, some recent studies reported that larger MMN amplitude was associated with higher ratings of attention to details, communication challenges, and sensory overresponsivity in NT and autistic children (Matsuba et al. 2024; Cary et al. 2023). This aligns with findings that both auditory sensitivity (Ben‐Sasson et al. 2009) and MMN amplitude decrease from childhood to adulthood in ASD. Interestingly, lower MMN amplitudes in autistic children and adolescents have been moderately associated with reduced global adaptive functioning, suggesting a link between MMN and inflexible behaviors in ASD (Lassen et al. 2022). Additionally, MMN amplitude correlates with behavioral discrimination ability (Näätänen et al. 2012), suggesting heightened sensory discrimination in autistic adults. This result is in line with theories of ASD suggesting increased sensory precision in ASD (Brock 2012; Mottron et al. 2006).

Within the predictive coding framework, ASD symptoms are hypothesized to arise from atypical perceptual inference or learning (Haker et al. 2016). Specifically, difficulties in building priors and downweighting prediction errors may impact perception, particularly altering the interpretation of inputs that are complex, dynamic, and noisy, such as those in social interactions. In this framework, sensory overresponsivity may result from inflexibly high prediction errors, while repetitive behaviors may serve as compensatory mechanisms to minimize prediction errors (Van de Cruys et al. 2014). In autistic adults, increased MMN amplitude in multifeature designs may reflect stronger prediction errors under uncertain conditions. In autistic children, reduced amplitude may reflect greater difficulty in learning statistical regularities in uncertain contexts. Importantly, despite group differences, the presence of MMN responses in autistic individuals indicates an ability to detect irregularities, suggesting that priors were learned.

### 4.4 Limitations

Several limitations should be acknowledged. First, in most of the analyses, residual heterogeneity remained even after accounting for moderators, highlighting additional unexplained variability. This may be due to differences in experimental paradigms (e.g., lack of counterbalanced designs), analysis methods (e.g., time window or reference electrode used), group heterogeneity (e.g., presence of comorbidities, broad age ranges), or evolving ASD diagnostic criteria over time (i.e., with changes from the DSM‐IV to DSM‐5 (American Psychiatric Association 2013)). These factors suggest that the certainty of some subgroup estimates may be moderate. Moreover, autistic individuals with intellectual disability are underrepresented in MMN studies, despite comprising approximately 38% of the autistic population (Maenner et al. 2020), restricting the applicability of these findings to the broader autism spectrum. Nonetheless, previous research suggests that intelligence quotient does not account for MMN differences between ASD and NT individuals (Schwartz et al. 2018). Furthermore, several studies were excluded from this meta‐analysis due to insufficient data reporting, emphasizing the need for improved standardization and transparency in reporting. Finally, while some studies have suggested that atypical MMN responses could serve as a biomarker for ASD, the heterogeneity of results and the presence of atypical MMN in other populations (Näätänen et al. 2012) indicate that atypical MMN is not specific to ASD, but may represent a broader marker of vulnerability to neurodevelopmental or psychiatric disorders.

## 5 Conclusions

In conclusion, this meta‐analysis demonstrated that autistic individuals exhibit MMN responses that are typical in some contexts but atypical in others. Measuring MMN amplitude and latency alone is often insufficient to differentiate ASD from NT individuals, highlighting the need for more nuanced paradigms combined with computational modeling to improve group differentiation. In multifeature designs, autistic adults showed larger MMN amplitudes, whereas autistic children and adolescents displayed reduced MMN amplitudes. These findings may reflect a developmental shift toward high and inflexible prediction errors in autistic adults (Van de Cruys et al. 2014). Paradigms relying on phoneme deviants also showed decreased MMN amplitudes in ASD. Importantly, while age group and certain experimental characteristics explained some variability, heterogeneity often remained unexplained. Longitudinal studies investigating MMN changes from childhood to adulthood in ASD could further shed light on the developmental trajectory of predictive processes. Additionally, investigating how inter‐individual differences relate to MMN in ASD could contribute to explaining the heterogeneous findings across studies. Finally, computational and fundamental approaches are crucial not only in ASD but also for other conditions where MMN is atypical, as they can provide deeper insights into its underlying learning and plasticity mechanisms.

## Conflicts of Interest

The authors declare no conflicts of interest.

## Supporting information


**Table S1:** PRISMA 2020 Checklist.
**Table S2:** Population characteristics of studies included in the meta‐analysis.

## Data Availability

The dataset is presented in Table 1, and further details are available from the corresponding author on request.
